# Pioneering a Neurosurgical Digital Subtraction Angiography (DSA) Program: Lessons From 100 Cases Using 3D C-arm Technology

**DOI:** 10.7759/cureus.104655

**Published:** 2026-03-04

**Authors:** Amarendra Pasham, Ramanadha Reddy Kanala, Suhas Malineni, Suchanda Bhattacharya, Sanjay Raj Kumar Reddy

**Affiliations:** 1 Neurosurgery, Nizam's Institute of Medical Sciences, Hyderabad, IND

**Keywords:** 3d c-arm technology neurosurgery operating theater, cerebrovascular diseases, digital subtraction angiography (dsa), endovascular procedures, hybrid neurosurgery

## Abstract

Introduction: Digital subtraction angiography (DSA) remains the gold standard for diagnosing and managing cerebrovascular diseases, despite the increasing use of noninvasive imaging modalities. Traditionally performed in catheterization labs by interventional radiologists, the integration of DSA into neurosurgery operating theaters (OT) using 3D C-arm technology offers neurosurgeons greater control over diagnosis and treatment planning, reducing dependency on external specialists.

Objective: This study aims to describe the initiation and implementation of a DSA program in a neurosurgery OT using 3D C-arm technology, detailing the technique, procedural nuances, and associated benefits. By sharing our institutional experience, we seek to provide a valuable resource for neurosurgeons interested in establishing similar programs.

Methods: Between October 2023 and January 2025, 100 diagnostic DSA procedures were performed in our neurosurgery OT utilizing a multi-axis 3D C-arm (Ziehm Vision RFD 3D, Ziehm Imaging, Germany). Patient selection criteria, procedural protocol, radiation safety measures, and complication management strategies were meticulously developed. Arterial access was obtained via the right femoral artery, and angiographic views were optimized to ensure comprehensive cerebrovascular evaluation. Post-procedural care included hemostasis, neurological monitoring, and complication prevention.

Results: Among the 100 DSA procedures performed, the most common indication was spontaneous subarachnoid hemorrhage (SAH) in 88 cases (88%). Two patients underwent intra-arterial nimodipine injection for vasospasm management post-aneurysm clipping. No major complications, including stroke or mortality, were encountered. Challenges such as radiation safety, staff training, and equipment acquisition were successfully addressed, leading to improved efficiency and procedural safety over time.

Conclusions: The successful implementation of a DSA program in the neurosurgery OT using 3D C-arm technology underscores its feasibility and benefits. Neurosurgeons, with their deep understanding of cerebrovascular anatomy and pathology, are well-positioned to perform DSA, manage complications, and advance endovascular capabilities. This initiative highlights the potential for hybrid neurosurgical practice, bridging the gap between open microsurgery and endovascular interventions, ultimately improving patient outcomes and advancing neurovascular care.

## Introduction

Digital subtraction angiography (DSA) remains a cornerstone in neurosurgery, facilitating the diagnosis and treatment of cerebrovascular diseases, including aneurysms, arteriovenous malformations (AVMs), and fistulas (AVFs), as well as CNS vasculitis and atherosclerotic vascular disease [1]. Although CT angiography has largely supplanted DSA for diagnostic purposes, DSA is still regarded as the gold standard investigation.

DSA is frequently reserved for treatment planning of endovascular or open surgical procedures when noninvasive imaging results are inconclusive or inconsistent. A key advantage of DSA is its real-time imaging capability, which surpasses that of noninvasive imaging modalities [2].

With the growing demand for neuro interventions, we firmly believe that neurosurgeons should possess basic knowledge of DSA and neuro interventions. The future of neurosurgery lies in developing hybrid neurosurgeons who can proficiently perform both microscopic and endovascular procedures [3]. Moreover, neurosurgeons are best equipped to manage complications arising from endovascular procedures.

By acquiring knowledge and skills in DSA, neurosurgeons can maintain greater control over the diagnostic and therapeutic process, reducing reliance on other specialties. Traditionally, DSA has been performed in a cath lab setting by interventionists. However, high-quality DSAs can be performed using 3D C-arm technology in a neurosurgery operating theater (OT) by neurosurgeons.

In our institution, we have successfully initiated a DSA program in the neurosurgery OT using 3D C-arm technology, completing over 100 diagnostic procedures to date. This achievement underscores the feasibility and effectiveness of performing DSA in a neurosurgery OT setting.

This article aims to provide a comprehensive description of our institutional protocol for performing DSA using 3D C-arm technology in a neurosurgery OT. The primary objectives are to delineate our technique of performing DSA using a 3D C-arm in a neurosurgery OT setting and to highlight the nuances and critical considerations involved in the technique, ensuring optimal image quality and patient safety. By sharing our experience and protocol, we aim to provide a valuable resource for neurosurgeons seeking to establish their DSA program.

We are describing two illustrative cases, one being an AVM and another a giant middle cerebral artery (MCA) aneurysm, along with the DSA images to depict the quality of DSA images in a 3D C-arm. This study describes a pioneering effort in integrating 3D C-arm-based DSA into a conventional neurosurgical operating room, redefining intraoperative imaging workflows and expanding real-time vascular assessment capabilities without reliance on a dedicated hybrid suite.

## Materials and methods

Study design

This study is a single-center, observational, descriptive analysis of the establishment and early experience of a DSA service within a neurosurgery OT. The study was conducted over a 16-month period from October 2023 to January 2025 at a tertiary-care government teaching hospital, Nizam's Institute of Medical Sciences, Hyderabad. The primary objective was to evaluate the feasibility, safety, and technical outcomes of performing cerebral angiography within the neurosurgery operating room using a mobile 3D C-arm system. Institutional approval was obtained prior to study initiation, and all procedures were performed in accordance with institutional ethical standards. Nizam's Institute of Medical Sciences issued approval NIMS/ISCR-1/436/24.

Angiography equipment and OT modification

All angiographic procedures were performed using a Ziehm Vision RFD 3D mobile C-arm system (Ziehm Imaging, Germany). The system was equipped with a flat-panel detector, multi-axis rotational capability, Linux-based 2D imaging software, and Windows-based 3D reconstruction software (Microsoft Corporation, United States). The imaging system allows real-time fluoroscopy, DSA, and 3D rotational angiography.

The neurosurgery OT was modified to accommodate angiographic procedures. A radiolucent Allen operating table (Baxter International, USA) was used to permit unobstructed fluoroscopic imaging and free C-arm movement. Radiation safety measures included the installation of lead-lined doors and shielding, restriction of unnecessary personnel during imaging, and adherence to institutional radiation safety protocols. All operating room staff involved in the procedures were trained in radiation protection practices.

Patient selection and indications

During the study period, 100 consecutive angiographic procedures were attempted in patients admitted under the neurosurgery service. Patient selection was based on clinical presentation and radiological findings. The most common indication for angiography was spontaneous subarachnoid hemorrhage (SAH), where DSA was performed for identification of an underlying vascular etiology. Additional indications included follow-up evaluation of moyamoya disease, suspected cerebral AVMs, and evaluation of selected cases of spontaneous intracerebral hemorrhage with no clear cause on non-contrast imaging.

The study represents the initial phase of program development; therefore, pediatric patients and complex endovascular therapeutic procedures were excluded. Procedures were categorized into diagnostic angiography and simple endovascular interventions, limited to intra-arterial vasodilator infusion for post-clipping vasospasm.

Pre-procedural preparation

All patients underwent standardized pre-procedural evaluation. Written informed consent was obtained from the patient or a legally authorized representative. Baseline investigations included complete blood counts, renal function tests, and coagulation profiles. Patients were kept nil per oral for at least six hours prior to the procedure. Adequate intravenous hydration was ensured. Antiplatelet and anticoagulant medications, when present, were withheld or managed according to institutional protocol. Both groins were prepared and draped to allow vascular access if required.

Anesthesia and monitoring

The majority of procedures were performed under local anesthesia, allowing continuous neurological monitoring during angiography. General anesthesia was used selectively in patients with poor neurological status, agitation, or inability to cooperate. Continuous monitoring of heart rate, blood pressure, oxygen saturation, and electrocardiography was maintained throughout the procedure.

**Figure 1:**
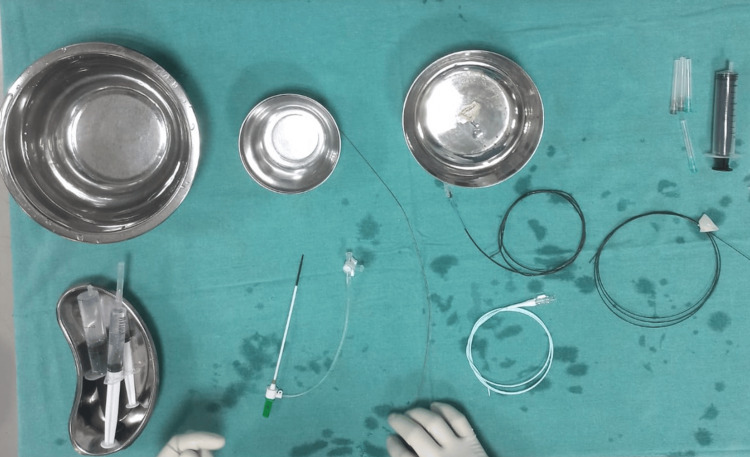
Operating trays and catheter system required for the diagnostic DSA, consisting of a short sheath, short wire, and gliding wire with a diagnostic catheter (H1). DSA: digital subtraction angiography

Basic instrumentation and tray setup are shown in Figure 1.

Vascular access and catheterization technique 

Vascular access was obtained via the right common femoral artery in all patients [4,5]. After local anesthetic infiltration, arterial puncture was performed using the Seldinger technique. A 5-French short arterial sheath was inserted, and its position was confirmed fluoroscopically. Following sheath placement, intravenous heparin (2500 units) was administered routinely to reduce thromboembolic risk.

Catheterization was performed using a wire-first technique under fluoroscopic guidance. Catheter selection was individualized based on patient age, aortic arch anatomy, and vessel tortuosity. Commonly used catheters included 5-French vertebral catheters, Judkins right coronary catheters in elderly patients with elongated arches, and Simmons or Headhunter catheters for complex arch anatomy. Navigation was facilitated using 0.035-inch hydrophilic guidewires.

After short sheath insertion, a diagnostic catheter with a gliding wire was introduced under fluoroscopic guidance. Using the "wire first" technique, the diagnostic catheter was navigated into the arch of the aorta. The wire was then removed, and the catheter was navigated into the cerebral vessels. It was essential to cannulate the right and left carotid and vertebral arteries and obtain anterior and posterior circulation images. A thorough understanding of aortic arch anatomy was crucial for easy navigation of catheters. Variations, such as a bovine arch, where the left carotid artery arises from the brachiocephalic trunk, should be recognized.

Angiographic protocol

Selective cannulation of the bilateral internal carotid arteries and vertebral arteries was attempted in all patients to achieve complete evaluation of both anterior and posterior circulations. Standard angiographic projections were obtained, including anteroposterior, lateral, oblique, and Towne’s views, depending on the vascular territory and suspected pathology. Contrast injection parameters were adjusted according to vessel size and flow characteristics.

In cases of suspected vasospasm following aneurysm clipping, intra-arterial nimodipine infusion was performed through a selectively positioned catheter under fluoroscopic control.

Angiographic views

**Figure 2:**
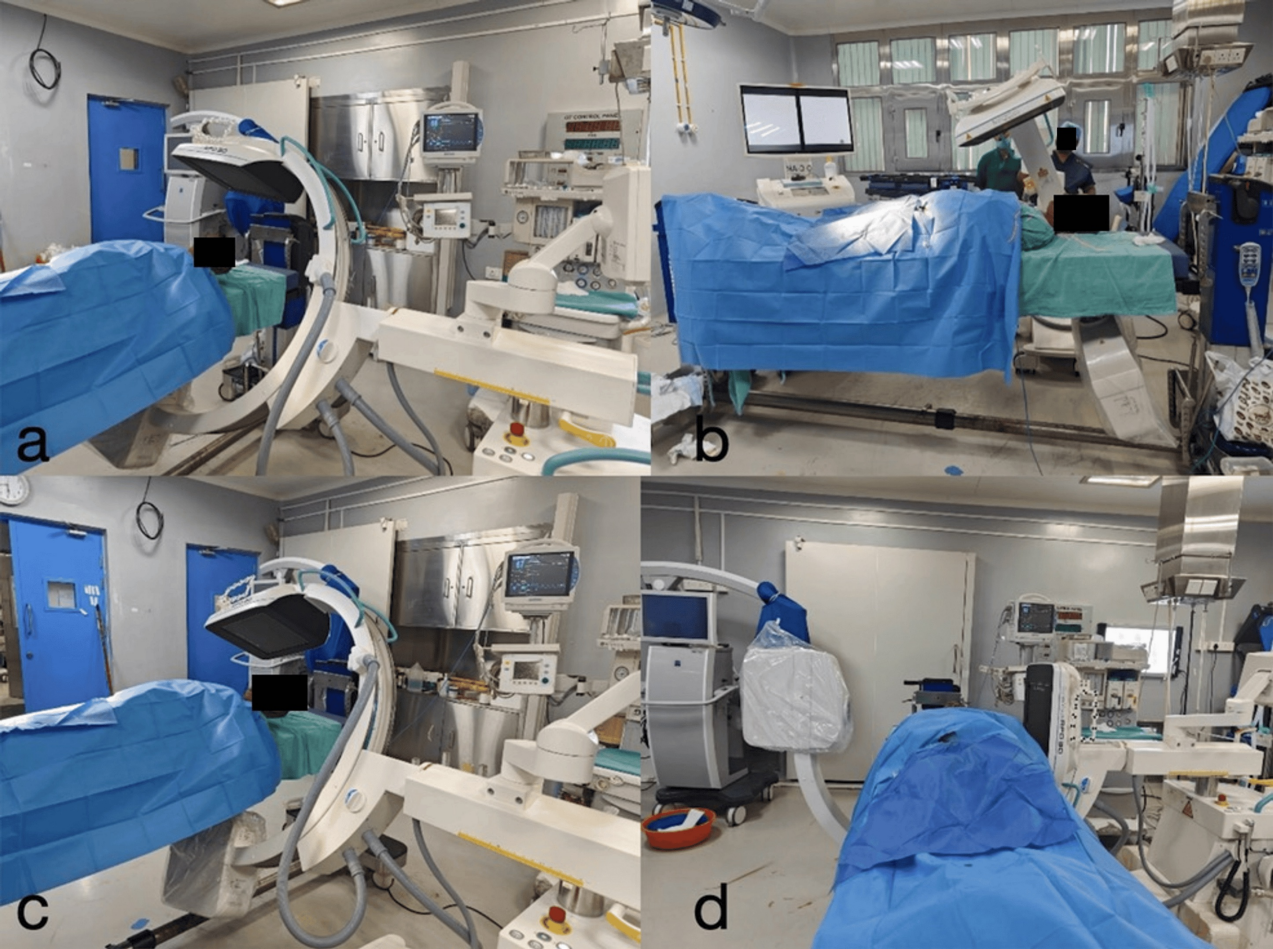
Hybrid operating room setup. This figure illustrates the reconstructed operating room using original photographs taken in the operating theater. a: Towne's view, this anteroposterior craniocaudal view, showcases the setup of the microscope and anesthesia machines; b-d: C-arm angulations.

To ensure a comprehensive evaluation of the cerebral vasculature, various angiographic views are employed, which are summarized in Table 1. The choice of view depends on the specific region of interest and the suspected pathology. Figure 2 shows the hybrid operating room setup with the state-of-the-art C-arm system and its various angulations.

**Table 1:** Specific angiographic views to view the anatomic structures. Data adapted from [6].

Anatomic Structure	Specific Angiographic Views
Carotid bifurcation	Posteroanterior (PA), oblique
Internal carotid artery (ICA)	
Cavernous and ophthalmic segments	Caldwell, lateral
Rest of the ICA	- Anteroposterior (AP) (0°) → supraclinoid ICA, MCA, and anterior cerebral artery (ACA) above bone margin
	- Lateral
	- Oblique (25-35°) → Assess ACA, ACOM, and MCA bifurcation
Posterior communicating artery	Lateral
Anterior communicating artery (ACOM)	- Submentovertical → Projects ACOM above the nasal cavity
	- Oblique (25-35°)
Middle cerebral artery (MCA)	Transorbital corresponding oblique
Vertebral artery and posterior cerebral artery (PCA)	- AP (20° caudal) → Petrous bone overlaps the inferior orbit margin, distal vertebral and basilar arteries in profile
	- Towne’s AP → PCA in profile
	- Lateral
Basilar artery	AP (20° caudal), lateral

Procedural challenges and abandoned procedures

In two patients, angiography was abandoned due to markedly complex and tortuous left carotid artery anatomy, which prevented safe catheter advancement despite multiple attempts and catheter exchanges. The decision to terminate the procedure was taken in the interest of patient safety. No access-site or neurological complications occurred in these patients. 

While rare, potential complications of DSA include groin hematoma (approximately 4% of cases), transient ischemic attack (2.5% of cases within 24 hours), stroke with permanent focal neurologic defect (0.1% of cases), and death (0.06% of cases) [7].

Radiation safety and dose optimization

Radiation exposure was minimized by adherence to As Low As Reasonably Achievable (ALARA) principles. Fluoroscopy time was kept to the minimum required for safe catheter navigation and image acquisition. Lead aprons, thyroid shields, and protective barriers were used by all staff. Radiation exposure parameters were monitored during procedures.

Post-procedural care and follow-up

After sheath removal, hemostasis was achieved using manual compression or vascular closure devices as appropriate. Patients were monitored in the intensive care unit with serial neurological examinations and access-site surveillance. Any new neurological deficit or access-site complication was documented and evaluated. Patients undergoing vasodilator infusion were closely monitored for hemodynamic stability.

Statistical analysis

Given the descriptive nature of the study, no inferential statistical testing was planned. Continuous variables were presented as mean values, while categorical variables were expressed as frequencies and percentages. Data were compiled and analyzed using standard spreadsheet-based software.

## Results

We have successfully performed 100 DSAs since initiating the program. The demographic factors and indications for DSA have been depicted in Table 2. Around 98 (98%) were in the first group, which is the diagnostic group, and two (2%) patients were in the simple endovascular group (who underwent chemical angioplasty and intra-arterial injection of nimodipine for vasospasm post aneurysm clipping). The demographic breakdown includes 48 (48%) male patients and 52 (52%) female patients with a mean age of 55 years. The most common indication was spontaneous SAH (88% of cases). Eight (8%) patients were follow-up cases of moyamoya, one case was left insular AVM (1%), and another was spontaneous bifrontal bleeds (1%). Notably, five patients (5%) presented with extremely tortuous left carotid arteries, rendering cannulation challenging. Aneurysm positivity was seen in 78 patients out of 88 patients (89%). DSA of spontaneous basi frontal bleed was negative. Fortunately, no post-procedural complications were encountered. The procedure was abandoned in two patients due to markedly complex and tortuous left carotid artery anatomy, precluding safe catheter navigation. No complications occurred in these patients. The angiographic findings of the DSA procedure are depicted in Table 3.

**Table 2:** Patient demographics and indications for DSA. DSA: digital subtraction angiography; AVM: arteriovenous malformation

Parameters	N = 100 (%)
Male	48 (48%)
Female	52 (52%)
Diagnostic DSA planned	98 (98%)
Simple endovascular procedure planned	2 (2%)
Spontaneous subarachnoid hemorrhage	88 (88%)
Moyamoya disease (follow-up)	8 (8%)
Cerebral AVM	1 (1%)
Spontaneous bifrontal hemorrhage	1 (1%)

**Table 3:** Angiographic findings (completed procedures). DSA: digital subtraction angiography; AVM: arteriovenous malformation; SAH: subarachnoid hemorrhage

DSA Findings	N = 100 (%)
Intracranial aneurysm among SAH	78 (78%)
Angiographically negative SAH	10 (10%)
Moyamoya disease	8 (8%)
Cerebral AVM	1 (1%)
No vascular pathology detected	3 (3%)

Among the 100 patients, intracranial aneurysms were most commonly located in the anterior communicating artery (ACOM) in 50 patients (50%), followed by the MCA in 20 patients (20%), the posterior communicating artery (PCoA) in six patients (6%), and the basilar bifurcation in two patients (2%). When analyzed among the aneurysm-positive cohort (n = 78), ACOM aneurysms accounted for 64.1% (n = 50), MCA aneurysms for 25.6% (n = 20), PCoA aneurysms for 7.7% (n = 6), and basilar bifurcation aneurysms for 2.6% (n = 2).

All moyamoya disease patients had previously undergone superficial temporal artery-MCA (STA-MCA) bypass and were evaluated with follow-up DSA to assess bypass patency. Good opacification of recipient vessels across the bypass was observed in all cases. 

One patient with spontaneous bilateral basifrontal bleed underwent DSA, which was negative for any intracranial vascular pathology. He was subsequently diagnosed with vasculitis and was managed accordingly. 

Case illustrations

Case 1

A 32-year-old male presented with a history of headache for two months. An initial CT scan of the brain showed no evidence of intracranial hemorrhage. A subsequent MRI of the brain demonstrated flow voids in the left insular region, suggestive of an AVM. The patient was therefore subjected to diagnostic DSA.

**Figure 3:**
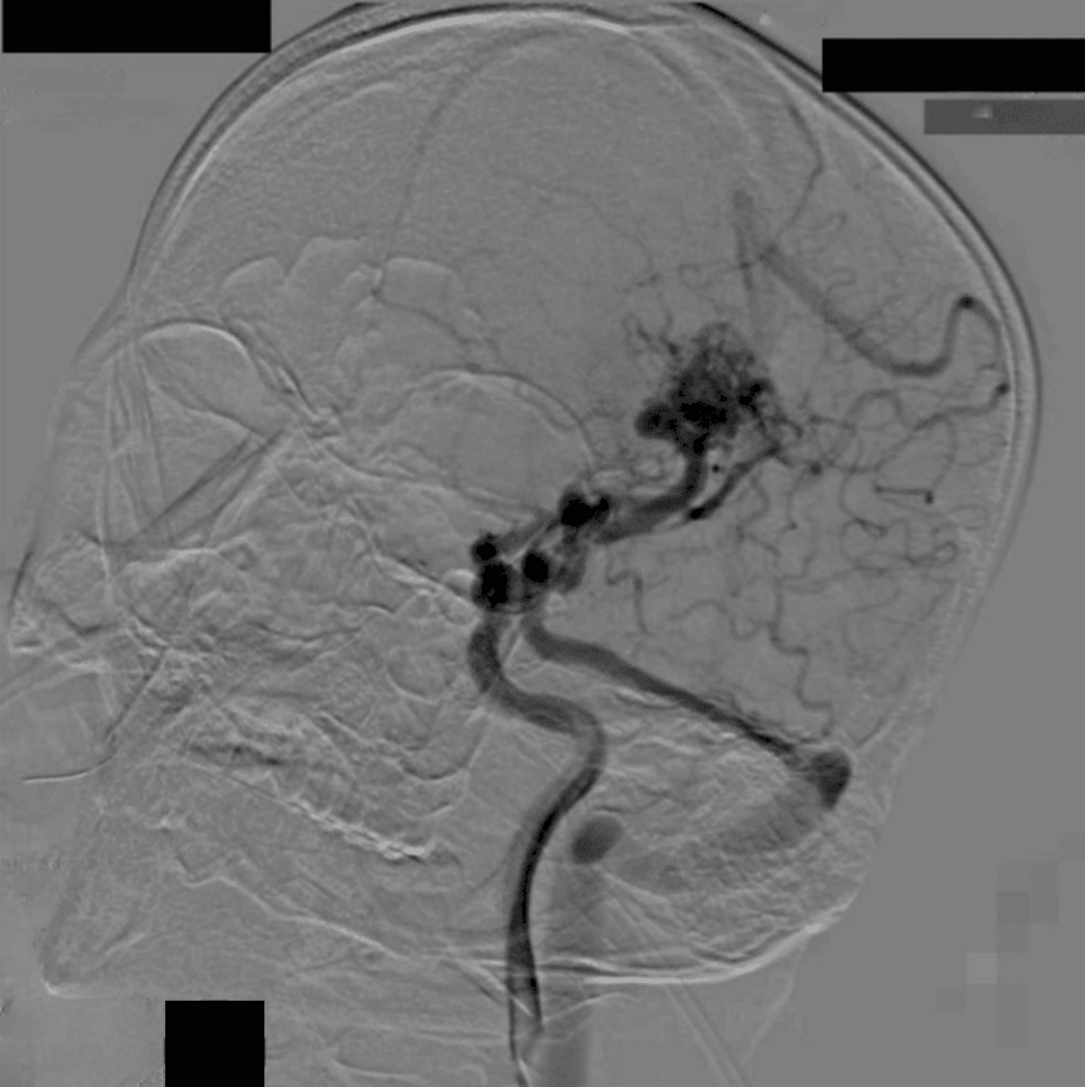
Oblique Towne’s view showing AVM nidus in the left insular region feeding from the MCA trunk and draining into the superior sagittal sinus and transverse sinus via the superior petrosal sinus. AVM: arteriovenous malformation; MCA: middle cerebral artery

DSA was performed using 3D C-arm acquisition, following the technical protocol described in the Materials and Methods section. Angiography revealed an AVM nidus measuring 2.1 × 1.9 × 2.2 cm, supplied predominantly by branches of the MCA trunk, with venous drainage into the superior sagittal sinus and transverse sinus, shown in Figure 3. Based on these findings, the patient was planned for stereotactic radiosurgery (SRS) with adjunctive endovascular embolization.

Case 2

A 45-year-old female presented to the emergency department with a history of headache for two days. Non-contrast CT (NCCT) of the brain demonstrated SAH localized to the left Sylvian fissure. Subsequent CT angiography revealed a giant aneurysm arising from the left MCA.

**Figure 4:**
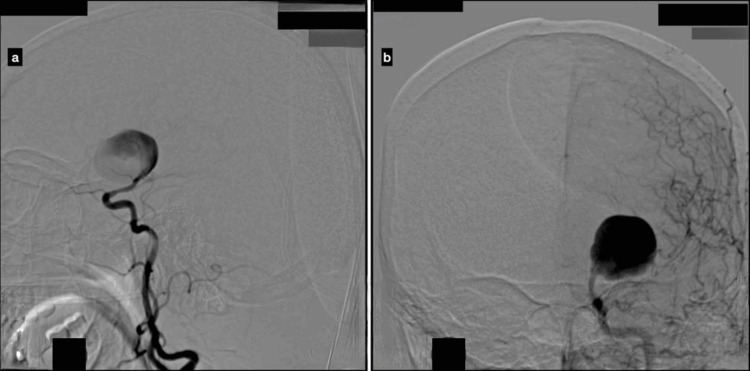
Lateral and Towne’s views of a patient who presented with symptoms of ICP and mass effect on NCCT of the brain. Left ICA injection showing a giant MCA aneurysm filling without opacification of the distal MCA branches. a: lateral view of the left ICA run; b: Towne's view of the left ICA run. ICA: internal carotid artery; MCA: middle cerebral artery; ICP: intracranial pressure; NCCT: non-contrast CT

For detailed vascular assessment, the patient underwent DSA. DSA confirmed a giant aneurysm originating from the left MCA trunk, visualized during the left internal carotid artery (ICA) run, shown in Figure 4. Distal MCA and anterior cerebral artery (ACA) branches were not visualized, likely due to preferential contrast filling of the giant aneurysm, resulting in poor opacification of the distal vessels. Based on these angiographic findings, the patient was planned for MCA trapping after STA-MCA bypass.

## Discussion

DSA remains the gold standard for the diagnosis and characterization of intracranial vascular pathologies. Traditionally, cerebral angiography and endovascular procedures have been performed in dedicated catheterization laboratories. However, in many resource-constrained settings, limited availability of cath lab time, logistical challenges in patient transfer, and the need for urgent vascular imaging in critically ill neurosurgical patients have driven interest in alternative solutions. The present study describes our experience in establishing a neurosurgery OT-based DSA program using a mobile 3D C-arm system and evaluates its feasibility, safety, and early outcomes.

The principal implication of our experience is that diagnostic cerebral angiography can be meaningfully integrated into a neurosurgery OT without compromising procedural integrity. Rather than serving merely as an alternative location, the OT-based platform reflects an evolution of the hybrid operating room concept, in which advanced imaging and surgical readiness coexist within a unified environment. Contemporary cerebrovascular literature increasingly supports hybrid models for complex aneurysm and vascular procedures, emphasizing their role in improving workflow continuity and enabling immediate surgical conversion when required [8].

In this context, the feasibility of OT-based angiography underscores the adaptability of modern multi-axis 3D C-arm systems, which have narrowed the technological gap between mobile platforms and fixed angiography suites. However, published experiences with hybrid ORs consistently highlight that infrastructure alone is insufficient; procedural success depends on structured radiation safety protocols, ergonomic equipment integration, and a trained multidisciplinary team familiar with both angiographic and operative demands. Our findings align with these reports and suggest that, when these prerequisites are met, the neurosurgical OT can extend beyond its traditional surgical role to become a functional cerebrovascular imaging environment.

Complex spinal surgeries have been performed using the radiolucent Allen table, allowing for the acquisition of axial and sagittal images [9,10]. The same setup has also been utilized for neurointerventional procedures. By adopting the hybrid operating room concept, we can perform complex spinal and neurointerventional procedures with greater precision, which enhances overall efficiency [11]. Findings indicate that these techniques contribute to accurate and effective surgical outcomes.

Reported aneurysm detection rates in spontaneous SAH in established catheterization laboratory series generally range between 75% and 85%, depending on the timing of angiography and patient selection. The diagnostic yield observed in our OT-based program falls within this reported range, suggesting that vascular opacification, spatial resolution, and projection adequacy achieved with a modern multi-axis 3D C-arm system are comparable to conventional fixed angiography suites. This comparison is clinically relevant, as the principal concern when transitioning angiography outside a cath lab environment is the potential for missed aneurysms and consequent rebleeding risk. The consistency of our detection rate with prior published series supports the interpretive conclusion that, when appropriate imaging protocols are followed, an OT-based DSA platform can maintain accepted diagnostic standards in the evaluation of spontaneous SAH, while angiographically negative cases remain in keeping with the recognized proportion of non-aneurysmal hemorrhage described in the literature.

Several recent reports of hybrid operating room and mobile C-arm angiography programs in neurosurgical practice describe a similar breadth of indications beyond aneurysmal SAH, including evaluation of moyamoya disease, AVMs, and other cerebrovascular pathologies. In these series, the ability of modern mobile imaging systems to depict collateral networks, stenotic segments, and nidus architecture has been generally comparable to that of fixed angiography suites when standardized imaging protocols are applied. Published experiences also emphasize the importance of high-quality projection angles and appropriate contrast timing to ensure diagnostic confidence for complex pathologies, reinforcing that image quality, rather than the physical setting, is the principal determinant of diagnostic utility. Furthermore, multiple centers report that the exclusion of treatable vascular lesions in selected intracerebral hemorrhage presentations is a valuable contribution of angiography to clinical decision-making. Our program’s range of indications and perceived imaging performance align with these observations, suggesting that, with adequate technical calibration and interpretive expertise, OT-based angiography can support a spectrum of neurovascular evaluations consistent with prior hybrid operating room and mobile C-arm literature.

A key strength of performing angiography within the neurosurgery OT lies in logistical efficiency and patient safety. Critically ill patients with SAH often require intensive monitoring, airway protection, and intracranial pressure management. Avoiding patient transfer to a remote cath lab reduces the risk of hemodynamic instability, accidental extubation, and delays in diagnosis. In addition, the proximity to the OT allows seamless transition to surgical intervention when required, particularly in cases of ruptured aneurysms.

In the present series, two procedures were abandoned due to markedly complex and tortuous left carotid artery anatomy that precluded safe catheter navigation. Importantly, the decision to abandon these procedures reflects a safety-first approach rather than a technical failure. No complications occurred in these patients. Such anatomical challenges are well described even in high-volume cath lab settings, and abandonment rates of a similar magnitude have been reported in conventional angiographic series. Including these cases transparently strengthens the validity of our results and highlights the importance of careful patient selection during the early phase of program development.

Another important observation from our experience was the learning curve associated with catheter selection and navigation. Early cases required longer fluoroscopy times and multiple catheter exchanges, particularly in elderly patients with elongated or tortuous aortic arches. With increasing operator experience, procedural efficiency improved, reflected by smoother catheterization and optimized imaging runs. Although fluoroscopy time and radiation dose were not formally quantified in this study, adherence to ALARA principles and radiation safety protocols ensured that exposure remained within acceptable limits for both patients and staff.

Radiation safety is a critical concern when performing angiography outside a conventional cath lab. In our setup, structural modifications to the OT, including radiation-resistant doors and shielding, along with strict staff training and protective measures, enabled safe conduct of procedures. The use of a radiolucent operating table and flexible C-arm positioning allowed optimal imaging without compromising surgical ergonomics. These considerations are essential for centers planning to adopt a similar model.

Only simple endovascular interventions were performed during the study period, limited to intra-arterial nimodipine infusion for post-clipping vasospasm. These procedures were completed without complications, demonstrating that basic therapeutic interventions can be safely incorporated into an OT-based angiography program. However, complex endovascular procedures were deliberately excluded during this initial phase, reflecting a cautious and staged approach to program expansion.

Limitations and future directions

This study has several limitations that should be considered when interpreting the findings. First, objective radiation exposure metrics for both patients and operating room personnel were not systematically recorded. As a result, conclusions regarding radiation safety are based on workflow observations rather than quantitative dose comparisons, limiting the ability to assess potential exposure reduction or increase relative to conventional environments. Future studies should incorporate standardized radiation dosimetry, including patient dose-area product and real-time staff exposure monitoring.

Second, the absence of a direct comparator group using a conventional catheterization laboratory or standard operating room restricts comparative evaluation of imaging quality, procedural efficiency, and workflow optimization. Without such a control, it is not possible to determine whether the observed advantages are attributable specifically to the hybrid operating room configuration. Prospective comparative studies or matched cohort analyses would help clarify the relative benefits and trade-offs of different procedural environments.

Third, this experience reflects a single-center implementation with a limited number of cases, which may limit generalizability to other institutions with differing infrastructure, case complexity, or operator experience. Multicenter studies with larger sample sizes would improve external validity and allow assessment of reproducibility across diverse practice settings.

Finally, the study did not evaluate clinical outcomes, cost-effectiveness, or long-term workflow efficiency. Future research should focus on outcome-based analyses, economic assessments, and structured workflow metrics to better define the role of hybrid operating rooms in complex neurovascular procedures.

## Conclusions

In conclusion, our experience demonstrates that establishing a neurosurgery OT-based DSA program using a mobile 3D C-arm system is feasible, safe, and diagnostically reliable. With appropriate infrastructure modification, adherence to radiation safety principles, and careful patient selection, high-quality cerebral angiography can be performed outside a conventional cath lab environment. This model offers a practical solution for centers facing cath lab constraints and may improve timely access to vascular imaging for neurosurgical patients.
